# Finding the precision to improve outcome in patients after cardiac arrest

**DOI:** 10.1186/s13054-017-1835-6

**Published:** 2017-10-22

**Authors:** Gentle Sunder Shrestha

**Affiliations:** Department of Anaesthesiology, Critical Care Unit, Tribhuvan University Teaching Hospital, Maharajgunj, Kathmandu, Nepal

In the trial by Kirkegaard et al [1], among the survivors of out-of-hospital cardiac arrest, Targeted temperature management (TTM) at 33 °C for 48 h, when compared with 24 h, did not improve neurological outcome at 6 months. Secondary injury following hypoxic ischemic brain injury after cardiac arrest involves multiple pathophysiologic derangements like free radical formation, release of excitatory neurotransmitters, endothelial dysfunction, intracellular accumulation of ionic calcium, etc. [2]. Multiple pathophysiologic mechanisms render these patients heterogeneous. TTM has been the mainstay of management in these patients to improve outcome. However, the existing trials have enrolled patients in a “one-size-fits all” approach. Future trials focusing not only on the timing, dose, and duration of TTM, but also on the various endotypes and targeting the other proposed pathopysiological mechanisms may help to explore potential therapeutic targets to improve outcome. Incorporating the information derived from genomic, proteomic, and metabolic data can help to indentify the various endotypes. The study by Kirkegaard et al. [1] was criticized for the study design, which was powered to detect 15% absolute difference in favorable outcome between groups. With that, the trial would not detect smaller differences in good outcome that can potentially alter clinical practice [3]. However, to attain a smaller effect size of 5%, a larger sample size of around 3000 patients would be necessary [1].

Registry-based randomized clinical trials, involving large clinical registries and enrolling unselected consecutive patients, could be a possible answer and the future direction of clinical trials. Such prospective trials would retain the strength of randomization and would permit enrollment of large numbers of patients while being cost-effective. It would also render the trials pragmatic and practical in real-world scenarios [4]. Considering the complex pathophysiology of hypoxic ischemic brain injury following cardiac arrest, multiple therapeutic interventions are theoretically possible. In the heterogeneous patient population, platform trials could be a plausible option to explore the effectiveness or futility of multiple treatments. It may also help to develop evidence bases to tailor treatment to subgroups defined by “omics” and biomarkers [5]. Together, registry-based randomized trials and platform trial design may help to pave a pathway towards precision in managing patients after cardiac arrest and thus to improve outcome.

## Abbreviation

TTM: Targeted temperature management
